# Age-Dependent Shift of AMPA Receptors From Synapses to Intracellular Compartments in Alzheimer’s Disease: Immunocytochemical Analysis of the CA1 Hippocampal Region in APP/PS1 Transgenic Mouse Model

**DOI:** 10.3389/fnagi.2020.577996

**Published:** 2020-10-06

**Authors:** Alejandro Martín-Belmonte, Carolina Aguado, Rocío Alfaro-Ruíz, Makoto Itakura, Ana Esther Moreno-Martínez, Luis de la Ossa, Elek Molnár, Yugo Fukazawa, Rafael Luján

**Affiliations:** ^1^Synaptic Structure Laboratory, Departamento de Ciencias Médicas, Facultad de Medicina, Instituto de Investigación en Discapacidades Neurológicas (IDINE), Universidad de Castilla-La Mancha, Albacete, Spain; ^2^Department of Biochemistry, Kitasato University School of Medicine, Sagamihara-shi, Japan; ^3^Departamento de Sistemas Informáticos, Escuela Superior de Ingeniería Informática, Universidad de Castilla-La Mancha, Albacete, Spain; ^4^School of Physiology, Pharmacology and Neuroscience, University of Bristol, Biomedical Sciences Building, Bristol, United Kingdom; ^5^Division of Brain Structure and Function, Faculty of Medical Sciences, Life Science Innovation Center, Research Center for Child Mental Development, University of Fukui, Fukui, Japan

**Keywords:** AD mouse model, hippocampus, electron microscopy, AMPA receptors, Alzheimer’s disease

## Abstract

Synapse loss occurs early in Alzheimer’s disease (AD) patients and animal models. Alterations at synaptic level are a major morphological correlate of the memory deficits and related symptoms of AD. Given the predominant roles of synaptic AMPA receptors (AMPARs) in excitatory synaptic transmission in the brain, changes in their dynamic regulation are also implicated in the pathophysiology of AD. Here, we used immunolocalization techniques to analyze the expression and subcellular distribution of AMPARs in the hippocampal region of APP/PS1 mouse model of AD. Immunoblots and histoblots revealed that the total amount of AMPARs and their regional expression pattern in the hippocampus was similar in APP/PS1 mice and in age-matched wild type mice. At the ultrastructural level, two synapse populations were examined using SDS-digested freeze-fracture replica labeling in the *stratum radiatum* in mice: (i) on spines of CA1 pyramidal cells; and (ii) on randomly found dendritic shafts of CA1 interneurons. While 1- and 6-months-old APP/PS1 mice exhibited no change, we observed a significant reduction at 12 months in AMPAR density at synapses in both pyramidal cells and interneurons, compared to wild-type. This reduction of AMPARs in dendritic spines was accompanied by a significant increase in AMPAR subunit proteins identified in intracellular compartments. Our data demonstrate an age-dependent reduction of synaptic AMPARs in APP/PS1 mice, which may contribute to impaired learning and memory at later stages of AD.

## Introduction

Alzheimer’s disease (AD) is a devastating neurodegenerative disorder characterized by progressive cognitive deficit and memory loss. Characteristic pathological features of AD are amyloid-β (Aβ) plaques and neurofibrillary tangles of aggregated hyperphosphorylated tau proteins in the brain (Bloom, 2014). Whilst this pathology is widespread throughout the brain, the entorhinal cortex and hippocampus are the most affected areas in AD (Hyman et al., 1984). In the hippocampus, CA1 pyramidal cells are the neurons most vulnerable to neurodegeneration, with a prominent loss of dendritic spines (West et al., 2000, 2004) and eventual cell death, but interneurons are also targeted in AD (Villette and Dutar, 2017). Soluble Aβ oligomers reduce the number of dendritic spines and impair glutamatergic synaptic transmission (Mucke and Selkoe, 2012).

Glutamatergic synaptic neurotransmission is regulated by the abundance and molecular composition of the glutamate receptor subtypes expressed at synapses by specific neuron types. The main targets of glutamatergic input in the hippocampus are the dendritic spines of pyramidal cells or granule cells and the dendritic shafts of interneurons (Spruston, 2008). Ionotropic glutamate receptors mediate the fast component of glutamatergic responses, mostly consisting of AMPA (α-amino-3-hydroxy-5-methyl-4-isoxazolepropionic acid) and NMDA (*N*-methyl-*D*-aspartate) receptors (Hollmann and Heinemann, 1994; Traynelis et al., 2010). AMPA receptors (AMPARs) are tetrameric complexes composed of homomeric or heteromeric combinations of four (GluA1–4) subunits, encoded by distinct genes, *GRIA1*–*GRIA4* (Traynelis et al., 2010). The majority of AMPARs in the hippocampus consist of heteromeric combinations of GluA1, GluA2, and GluA3 subunits (Keinänen et al., 1990). Immunoelectron microscopy using post-embedding immunogold labeling has been used to investigate AMPARs at excitatory synapses in the hippocampus in normal brains (Nusser et al., 1998; Petralia et al., 1999; Takumi et al., 1999; Racca et al., 2000). However, until now, these high-resolution techniques have not been applied for the identification of AD-related pathological changes in AMPAR numbers and densities.

Given the central role of AMPARs in learning and memory, their disfunction likely contribute to synaptic and memory deficits associated with AD. Consistent with this idea, previous studies have shown that Aβ can contribute to the down-regulation of synaptic transmission as a consequence of AMPAR internalization (Almeida et al., 2005; Hsieh et al., 2006; Gu et al., 2009; *but see* Whitcomb et al., 2015). Previous studies using autoradiography, *in situ* hybridization, immunoblots or immunohistochemical techniques produced conflicting results regarding the expression of AMPARs in AD brains. Some studies have shown that the distributions of subunits (GluA1, GluA2/3, GluA4) in the hippocampus of AD brains are similar to control brains (Hyman et al., 1994). In contrast, other studies suggested that GluA2 and GluA2/3 are reduced, but GluA1 is unchanged (Carter et al., 2004). Furthermore, earlier studies also proposed a reduction in GluA1 levels (Pellegrini-Giampietro et al., 1994), or consistent decrease in all four AMPAR subunits (Ikonomovic et al., 1995, 1997; Aronica et al., 1997; Thorns et al., 1997; Wakabayashi et al., 1999). However, these changes were not investigated at the level of individual neurons or synapses.

At present, it is not clear whether AMPARs are altered at all excitatory synapses in the hippocampus of AD brains. They may also be selectively disrupted at specific postsynaptic sites or neuron types. To clarify these possibilities in the hippocampal CA1 region, we employed immunoblots, histoblots, and high-resolution quantitative immunocytochemical techniques, with specific focus on the hippocampal regions of APP/PS1 mice. Here we provide compelling new evidence for a reduction in synaptic AMPARs in pyramidal cells and interneurons of the hippocampal CA1 region in the APP/PS1 AD mouse model, with a parallel increase in intracellular AMPAR population.

## Materials and Methods

### Animals

Male APP/PS1 mice (RRID:IMSR_MMRRC:034832) were obtained from the Jackson Laboratory^1^ and expressed Mo/Hu APP695swe construct in conjunction with the exon-9-deleted variant of human presenilin 1 [Tg(APPswe,PSEN1dE9)85Dbo/Mmjax] (Jankowsky et al., 2001, 2004). The “control” wild type (WT) mice were age-matched littermates without the transgene. The following ages were selected for analysis: (i) no sign of pathology (1 month), (ii) first signs of Aβ deposition (6 months) (Jankowsky et al., 2004), and (iii) onset of memory deficits with severe synapse loss and widespread Aβ deposition (12 months) (Garcia-Alloza et al., 2006; Gimbel et al., 2010). For all ages and genotypes, mice were used as follows for the experiments: Immunoblot (4), Histoblot (4), SDS-digested freeze-fracture replica labeling (SDS-FRL) (4) and pre-embedding immunogold experiments (3). All mice were maintained at the Animal House Facility of the University of Castilla-La Mancha (Albacete, Spain) in cages of two or more mice, on a 12 h light/12 h dark cycle at 24°C and received food and water *ad libitum*. Care and handling of animals prior to and during experimental procedures were in accordance with Spanish (RD 1201/2015) and European Union regulations (86/609/EC), and all protocols and methodologies were approved by the local Animal Care and Use Committee.

For immunoblotting and histoblotting, animals were deeply anesthetized by intraperitoneal injection of ketamine/xylazine 1:1 (ketamine, 100 mg/Kg; xylazine, 10 mg/Kg), the hippocampus was dissected, frozen rapidly in liquid nitrogen and stored at −80°C. For immunohistochemistry experiments at both the light microscopic and electron microscopic level, using the pre-embedding immunogold technique, animals were firstly deeply anesthetized by intraperitoneal injection of ketamine-xylazine 1:1 (ketamine, 100 mg/Kg; xylazine, 10 mg/Kg) and then transcardially perfused with ice-cold fixative containing 4% (w/v) paraformaldehyde with 0.05% (v/v) glutaraldehyde in 0.1 M phosphate buffer (PB, pH 7.4) for 15 min. After perfusion, brains were removed and immersed in the same fixative for 2 h or overnight at 4°C. Tissue blocks were washed thoroughly in 0.1 M PB. Coronal sections (60 μm thickness) were cut using a Vibratome (Leica V1000). For SDS-FRL, see below.

### Antibodies and Chemicals

For SDS-FRL, we used a new in-house generated rabbit anti-GluA1–4 (pan-AMPA) receptor polyclonal antibody (D160) (Supplementary Figure 1), whose preparation and purification were carried out by following and updating protocols used to prepare a previous in-house rabbit pan-AMPAR antibody (Nusser et al., 1998; Pickard et al., 2000). In brief, the antibody was raised against a glutathione *S*-transferase (GST) fusion protein that contained the 58 extracellular amino-acid residues (724–781) that preceded the last membrane-spanning segment of GluR1*flop* [GSTGluA1flop_(724–781)_] and affinity purified with a maltose-binding protein fusion protein with the identical amino acid residues used for immunization. The aa sequence is highly conserved in GluA1–4 and therefore the obtained antibody is expected to react with all AMPAR subunit proteins (Pickard et al., 2000; Supplementary Figure 1).

For histoblot and immunoblots, we used a guinea pig pan-GluA1–4 receptor antibody (GP-Af580; aa. 717–754 of mouse AMPA; Frontier Institute Co., Japan). The antibody characteristics have been described previously (Fukaya et al., 2006). For the pre-embedding immunogold technique, we used a polyclonal rabbit antibody anti-GluA2/3 (AB1506; Chemicon, Temecula, CA, United States). These antibodies were raised against synthetic peptides derived from intracellular C-terminal sequences of the GluA2 and GluA3 subunits (Wenthold et al., 1992). A monoclonal anti-α-tubulin antibody (DM1A; ref CP06) was obtained from Millipore (Millipore Corporation, Burlington, MA, United States). For double-SDS-FRL, we used a mouse monoclonal antibody against the GluN1 subunit of NMDA receptor (MAB363, Millipore Bioscience Research Reagents). The characteristics and specificity of GluN1 was characterized previously (Siegel et al., 1994; Iwasato et al., 2000; Masugi-Tokita et al., 2007; Szabadits et al., 2011). While the guinea pig AMPAR antibody worked well for histoblots and immunoblots, it produced relatively weak labeling in SDS-FRL. Therefore, we used rabbit anti-GluA1–4 antibodies for SDS-FRL.

The following secondary antibodies were used: goat anti-mouse IgG-horseradish peroxidase (1: 2,000; Santa Cruz Biotechnology, Santa Cruz, CA, United States), goat anti-rabbit IgG-horseradish peroxidase (1: 15,000; Pierce, Rockford, United States), alkaline phosphatase (AP)-goat anti-mouse IgG (H + L) and AP-goat anti-rabbit IgG (H + L) (1: 5,000; Invitrogen, Paisley, United Kingdom), anti-rabbit IgG conjugated to 10 nm gold particles and anti-mouse IgG conjugated to 5 nm gold particles (1: 100; British Biocell International, Cardiff, United Kingdom).

### Immunoblots

Hippocampi were homogenized in 50 mM Tris Base, pH 7.4, and protease inhibitor cocktail (Thermo Fisher Scientific, Pierce, Rockford, United States) with a motorized pestle (Sigma-Aldrich). The homogenized tissue was initially centrifuged 10 min at 1,000 × *g* at 4°C and the supernatant was further centrifuged 30 min at 12,000 × *g* (Centrifuge 5415R, Eppendorf, Hamburg, Germany) at 4°C. The resulting pellet, containing the membrane extracts, was resuspended in 50 mM Tris-Cl, pH 7.4, and protease inhibitor cocktail (Thermo Fisher Scientific, Pierce, Rockford, United States). The protein content of each membrane extract was determined by the BCA protein assay kit (Thermo Fisher Scientific). Twenty five micrograms of membrane protein was loaded onto sodium dodecyl sulfate polyacrylamide (7.5%) gels (SDS/PAGE) in sample buffer [0.05 M Tris pH 6.8, 2% (w/v) SDS, 10% (v/v) glycerol, 0.05% (v/v) β-mercaptoethanol, and 0.001% (w/v) bromophenol blue]. The proteins were transferred to PVDF membranes using a semidry transfer system, followed by immunolabeling with anti-GluA1–4 (1:1,000) and anti-α-tubulin (1:1,000) antibodies. Protein bands were visualized and detected after application of a mouse IgG kappa binding protein coupled to horseradish peroxidase (1:2,000 dilution) and using the enhanced chemiluminescence (ECL) blotting detection kit (SuperSignal West Dura Extended Duration Substrate, Pierce, Rockford, United States). Blots were captured and quantified by densitometry using a LAS4000 MINI (Fujifilm, Japan).

### Histoblotting

The regional distribution of AMPARs was analyzed in rodent brains, using the histoblot technique (Molnár, 2016; Aguado and Luján, 2019). Briefly, horizontal cryostat sections (10 μm) from mouse brain were overlayed with nitrocellulose membranes moistened with 48 mM Tris-base, 39 mM glycine, 2% (w/v) sodium dodecyl sulfate and 20% (v/v) methanol for 15 min at room temperature (∼20°C). After blocking in 5% (w/v) non-fat dry milk in phosphate-buffered saline for 1 h, nitrocellulose membranes were treated with DNase I (5 U/mL), washed and incubated in 2% (w/v) sodium dodecyl sulfate and 100 mM β-mercaptoethanol in 100 mM Tris–HCl (pH 7.0) for 60 min at 45°C to remove any remaining tissue residues. After extensive washing, the blots were reacted with affinity-purified anti-GluA1–4 antibodies (0.5 mg/mL) in blocking solution overnight at 4°C. The bound primary antibodies were detected with alkaline phosphatase-conjugated anti-rabbit IgG secondary antibodies (Molnár, 2016; Aguado and Luján, 2019). To compare the expression levels of AMPARs between the wild type and APP/PS1 mice and at all ages, all nitrocellulose membranes were processed in parallel, and with the same incubation time for each reagent was used for the antibody. Digital images were acquired by scanning the nitrocellulose membranes using a desktop scanner (HP Scanjet 8300). Image analysis and processing were performed using the Adobe Photoshop software (Adobe Systems, San Jose, CA, United States) as described previously (Martín-Belmonte et al., 2019). For both, a series of primary and secondary antibody dilutions and incubation times were used to optimize the experimental conditions for the linear sensitivity range of all reactions and to confirm that all labeling was below saturation levels.

### Immunohistochemistry for Electron Microscopy

Immunohistochemical reactions at the electron microscopic level were carried out using the pre-embedding immunogold and the SDS-FRL methods as described earlier (Tanaka et al., 2005; Luján et al., 2018).

#### Pre-embedding Immunogold Method

Briefly, free-floating sections obtained from WT and APP/PS1 and three ages (1, 6, and 12-months) were incubated in parallel in 10% (v/v) NGS diluted in TBS. Sections were then incubated in anti-GluA2/3 antibodies (3–5 μg/mL diluted in TBS containing 1% (v/v) NGS), followed by incubation in goat anti-rabbit IgG coupled to 1.4 nm gold (Nanoprobes Inc., Stony Brook, NY, United States). Sections were post-fixed in 1% (v/v) glutaraldehyde and washed in double-distilled water, followed by silver enhancement of the gold particles with an HQ Silver kit (Nanoprobes Inc.). Sections were then treated with osmium tetroxide [1% (w/v) in 0.1 M phosphate buffer], block-stained with uranyl acetate, dehydrated in graded series of ethanol and flat-embedded on glass slides in Durcupan (Fluka) resin. Regions of interest were cut at 70–90 nm using an ultramicrotome (Reichert Ultracut E, Leica, Austria) and collected on single slot pioloform-coated copper grids. Ultrastructural analyses were performed in a JEOL-1010 electron microscope.

#### SDS-FRL Technique

Animals were anesthetized with sodium pentobarbital (50 mg/kg, i.p.) and perfused transcardially with 25 mM PBS for 1 min, followed by perfusion with ice-cold fixative containing 2% (w/v) paraformaldehyde in 0.1 M phosphate buffer (PB) for 12 min. After perfusion, brains were immediately removed from the skull, and then the hippocampi were dissected and cut into coronal slices (130 μm) using a Microslicer (Dosaka, Kyoto, Japan) in 0.1 M PB. Next, hippocampal slices containing the CA1 region were trimmed out of the slices, and immersed in graded glycerol of 10–30% (v/v) in 0.1 M PB at 4°C overnight. Slices were frozen using a high-pressure freezing machine (HPM010, BAL-TEC, Balzers). Slices were then fractured into two parts at −120°C and replicated by carbon deposition (5 nm thick), platinum (60° unidirectional from horizontal level, 2 nm), and carbon (15–20 nm) in a freeze-fracture replica machine (BAF060, BAL-TEC, Balzers). Replicas were transferred to 2.5% (w/v) SDS and 20% (w/v) sucrose in 15 mM Tris-Cl buffer (pH 8.3) for 18 h at 80°C with shaking to dissolve tissue debris. The replicas were washed three times in 50 mM Tris-buffered saline (TBS, pH 7.4), containing 0.05% (w/v) bovine serum albumin (BSA), and then blocked with 5% (w/v) BSA in the washing buffer for 1 h at room temperature. Next, the replicas were washed and reacted with the following primary antibodies: (1) pan-GluA1–4 antibody raised in rabbit (7.3 μg/mL), or (2) mixture of the pan-GluA1–4 and mouse monoclonal antibody against the GluN1 subunit of NMDA receptor (10 μg/mL; Millipore Bioscience Research Reagents), diluted in 25 mM TBS containing 1% (w/v) BSA overnight at 15°C. Following three washes in 0.05% BSA in TBS and blocking in 5% (w/v) BSA/TBS, replicas were incubated in goat anti-rabbit (for pan-GluA1–4) IgGs coupled to 5 nm gold particles (1:30; British BioCell Research Laboratories) diluted in 25 mM TBS containing 5% (w/v) BSA overnight at room temperature. In the double-labeling protocols, replicas were first reacted with the pan-GluA1–4 antibody and then anti-rabbit secondary antibody conjugated to 10 nm gold particle, followed by incubation with the GluN1 antibody and then anti-mouse secondary antibody conjugated to 5 nm gold particle. When the primary antibody was omitted, no immunoreactivity was observed. After immunogold labeling, the replicas were immediately rinsed three times with 0.05% BSA in TBS, washed twice with distilled water, and picked up onto grids coated with pioloform (Agar Scientific, Stansted, Essex, United Kingdom) and examined with an electron microscope (Hitach H-7650) equipped with a digital camera (Quemesa, EM SIS).

### Quantification and Analysis of SDS-FRL Data

The labeled replicas were examined using a transmission electron microscope (JEOL-1010) and images captured at magnifications of 80,000× or 100,000×. All antibodies used in this study were visualized by immunoparticles on the exoplasmic face (E-face), consistent with the extracellular location of their epitopes. Non-specific background labeling for anti-AMPAR was estimated by counting immunogold particles on the protoplasmic face (P-face) surfaces in wild type mice. This value was on average 2.3 immunoparticles/μm^2^, and was not subtracted from values for specific labeling given the low value. Digitized images were then modified for brightness and contrast using Adobe PhotoShop CS5 (Mountain View, CA, United States) to optimize them for quantitative analysis.

#### Number and Density of AMPAR Immunoparticles in Excitatory Synapses

We determined the number of AMPAR immunoparticles at excitatory synapses present in dendritic spines of CA1 pyramidal cells in the *stratum radiatum* and in the dendritic shafts of interneurons located in the *stratum radiatum* of the CA1 region of the hippocampus, in both wild type and APP/PS1 mice and at all three ages. For this aim, we used the software GPDQ (*Gold Particle Detection and Quantification*) developed recently to perform automated and semi-automated detection of gold particles present in a given compartment of neurons (Luján et al., 2018). The vast majority of the spines in *stratum radiatum* arise from pyramidal cells, thus we refer to them as pyramidal cell spines. Dendritic shafts receiving multiple excitatory and inhibitory synapses are considered to originate from interneurons. For identification of neuronal compartment in the SDS-FRL samples, oblique dendrites were identified based on their small diameter and the presence of at least one emerging spine from the dendritic shaft. Dendritic spines were considered as such if: (i) they emerged from a dendritic shaft, or (ii) they opposed an axon terminal. Dendritic spines are smaller in size compared to dendritic shafts of interneurons. Given these differences in size, excitatory synapses in spines are normally observed with a concave shape, while in interneurons they have a more flattened morphology. Axon terminals were identified based on: (i) the presence of an active zone (AZ) facing a postsynaptic density (PSD), recognized by an accumulation of intramembrane particles (IMPs), on the opposing exoplasmic-face (E-face) of a spine or dendrite; or (ii) the presence of synaptic vesicles on their cross-fractured portions. Non-specific background labeling was measured on P-face structures surrounding the measured E-faces.

Quantitative analysis of immunogold labeling for AMPAR subunits was performed on excitatory postsynaptic specializations indicated by the presence of intramembrane particle (IMP) clusters on the exoplasmic face (E-face) (Harris and Landis, 1986). One of the advantages of the SDS-FRL technique is that the whole synaptic specialization of excitatory synapses is immediately visible over the surface of neurons. The outline of postsynaptic specialization (IMP clusters) is manually demarcated by connecting the outermost IMP particles, and the area of synaptic sites is measured using the software GPDQ. Immunogold particles for AMPARs were regarded as synaptic labeling if they were within demarcated IMP clusters and those located in the immediate vicinity within 30 nm from the edge of the IMP clusters, i.e., the potential distance between the immunogold particles and antigens. The number of immunogold particles was counted in both complete and incomplete (partially fractured) postsynaptic membrane specialization. An incomplete postsynaptic membrane specialization was considered as such when it was partially cut by a fracture, but contained at least 30 IMPs. AMPAR-positive excitatory synapses that were co-labeled with more than two immunoparticles for GluN1 were included as synaptic IMP clusters in the analysis to make sure we were dealing with glutamatergic synaptic contacts. As densities of immunogold labeling for the pan-GluA1–4 antibody obtained from complete and incomplete synapses were not significantly different, they were pooled. The density of the immunoparticles for AMPARs in each synaptic site was calculated by dividing the number of the immunoparticles by the area of the demarcated IMP clusters. Measurements were performed in three animals, and results were pooled due to the fact that the density for immunogold particles was not significantly different in the different animals. Then, we calculated the average density across synapses. Immunoparticle densities are presented as mean ± SEM.

Finally, it is worth mentioning that neuronal loss is only observed adjacent to plaques in the APP/PS1 mouse model^2^. Accordingly, our quantitative analysis was performed in Aβ plaque-free regions of the hippocampus to avoid the destroyed tissue in dystrophic neurites adjacent to Aβ plaques (Alonso-Nanclares et al., 2013), and also carried out systematically on identified excitatory synapses, which were similar in numbers (n) and total surface areas between the two genotypes. Thus, the density values expressed as immunoparticles/μm^2^ in the APP/PS1 mice at 12 months of age represent genuine reduction of AMPARs in different types of CA1 excitatory synapses, regardless of any possible neuronal and/or synaptic loss.

### Controls

To test method specificity in the procedures for electron microscopy, the primary antibodies were either omitted or replaced with 5% (v/v) normal serum of the species of the primary antibody, resulting in total loss of the signal. For the pre-embedding technique, labeling patterns were also compared with those obtained by calbindin (polyclonal rabbit anti-calbindin D-9k CB9; Swant, Marly, Switzerland); only the antibodies against GluA1–4 consistently labeled excitatory synapses.

### Data Analysis

To avoid observer bias, we performed blinded experiments for immunoblots and immohistochemistry prior to data analysis. Statistical analyses were performed using GraphPad Prism (San Diego, CA, United States) and data were presented as mean ± SEM unless indicated otherwise. Statistical significance was defined as *p* < 0.05. The statistical evaluation of the immunoblots was performed using the Student-Fisher *t*-test, with Levene’s test for homogeneity of variance and Shapiro-Wilks normality test. The application of Levene’s test indicated that variances are equal, and the application of Shapiro-Wilks test indicated that distributions were normal. To compute SEM error bars, five blots were measured from each animal. The statistical evaluation of the immunogold densities in the mouse model was performed using the two-way ANOVA test and Bonferroni *post-hoc* test. Correlations were assessed using Pearson’s correlation test. Finally, statistical evaluation of the frequency of immunogold measured with pre-embedding and frequency of co-localization measured with immunofluorescence were performed using student-t test with Holm-Sidak correction, following the application of Shapiro-Wilks test indicating that distributions were normal.

## Results

### AMPAR Expression Patterns Are Similar in Control and APP/PS1 Mice

**FIGURE 1:**
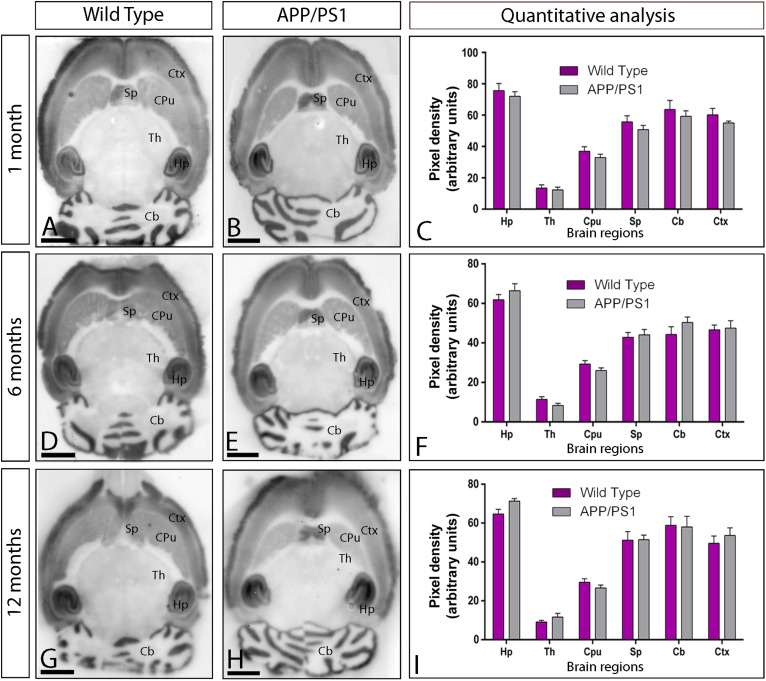
Regional expression of AMPARs in the brain in wild type and APP/PS1 mice. **(A–I)** Expression of GluA1–4 proteins was visualized using histoblots of horizontal brain sections at 1, 6, and 12 months of age in wild type and APP/PS1 mice, using an affinity-purified anti-GluA1–4 antibody. AMPAR expression in different brain regions was determined by densitometric analysis of the scanned histoblots **(C,F,I)**. The highest level of AMPAR expression was detected in the hippocampus (Hp), followed by the cerebellum (Cb), cortex (Cx), and septum (Sp). Lower expression levels were detected in the caudate putamen (CPu) and the thalamus (Th). Densitometric analysis showed no differences in AMPAR expression in APP/PS1 mice compared to age-matched wild type controls. Error bars indicate SEM. Scale bars: 0.2 cm.

Using the guinea pig anti-GluA1–4 antibody in conventional histoblots (Aguado and Luján, 2019), we determined in first place whether the expression of AMPAR was altered in the brain of APP/PS1 mice at different ages: 1 month (Figures 1A–C), 6 months (Figures 1D–F), and 12 months (Figures 1G–I). The overall expression patterns of GluA1–4 was very similar in wild type and APP/PS1 mice (Figures 1A–I). In wild type mice at all three ages, the strongest GluA1–4 immunoreactivity was found in the neuropil layers of the hippocampal formation, in the superficial layers of neocortex, and in the cerebellar molecular layer (Figures 1A,D,G). Moderate labeling was found in deeper layers of the neocortex, in the caudate putamen and thalamus (Figures 1A,D,G). This expression pattern was very similar in the brain of APP/PS1 mice at all ages (Figures 1B,E,H). Quantitative analyses performed to compare the protein densities for all ages revealed that GluA1–4 immunoreactivities were similar between wild type and APP/PS1 mice (Figures 1C,F,I).

**FIGURE 2:**
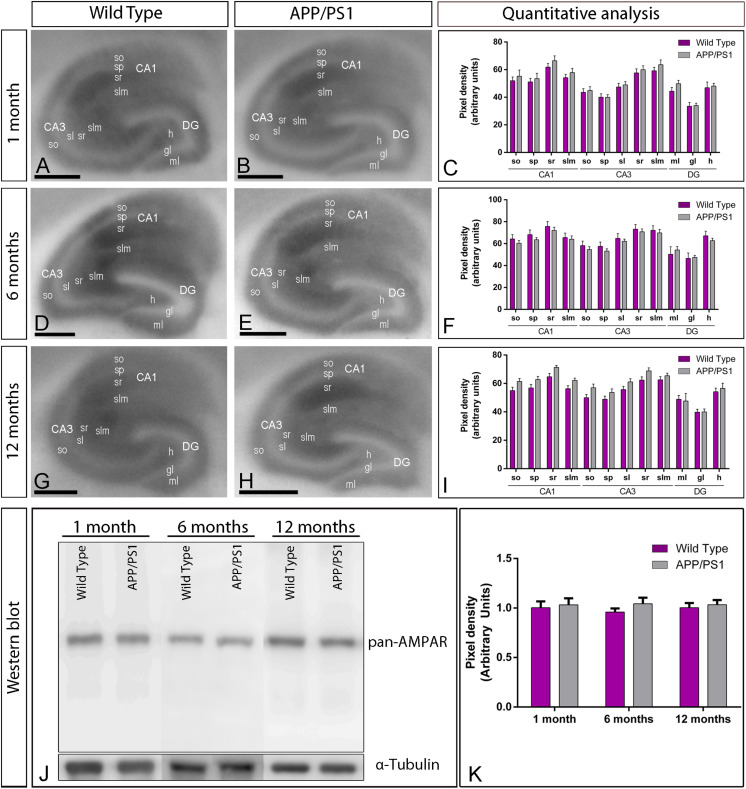
Hippocampal expression and distribution of AMPARs in wild type and APP/PS1 mice. **(A–I)** The expression of GluA1–4 proteins was visualized in histoblots of horizontal brain sections at 1, 6, and 12 months of age in wild type and APP/PS1 mice using an affinity-purified anti-GluA1–4 antibody. The level of AMPAR immunoreactivities in different hippocampal subfields and dendritic layers were determined by densitometric analysis of scanned histoblots. AMPAR expression was moderate to strong in all dendritic layers of the CA1 and CA3 region and dentate gyrus, with the *stratum radiatum* (sr) of CA1 and CA3 showing the highest expression level. Densitometric analysis showed no differences in AMPAR expression in APP/PS1 mice compared to age-matched wild type controls. Error bars indicate SEM. Scale bars: 0.05 cm. **(J,K)** Immunoblots showing the expression of the AMPAR protein in the hippocampus at 1, 6, and 12 months of age in wild type and APP/PS1 mice. Crude membrane preparations were probed with the anti-GluA1–4 antibody, which recognized a band with estimated molecular mass of 100 kDa. The developed immunoblots were scanned and densitometric measurements from five independent experiments (*n* = 4 brains) were averaged to compare the protein densities for each age and experimental group. AMPAR immunoreactivities were normalized to the α-tubulin content of each sample and expressed as pixel density. No differences were detected in APP/PS1 mice compared to age-matched wild type controls. Error bars indicate SEM.

Given that one of the most vulnerable brain regions affected in AD is the hippocampus (West et al., 2000, 2004), we next focused on this brain region to explore the laminar expression pattern of AMPARs. GluA1–4 is widely expressed in all hippocampal regions and dendritic layers at all ages of both wild type and APP/PS1 mice (Figures 2A–I). In the CA1 and CA3 regions of wild type mice, at all ages, GluA1–4 expression was moderate to strong, with the *strata oriens* and *lacunosum-moleculare* showing the lowest and the *stratum radiatum* showing the highest expression levels (Figures 2A,D,G). The *stratum lucidum* in the CA3 region showed weak expression level throughout (Figures 2A,D,G). In the dentate gyrus of wild type mice, GluA1–4 immunolabeling was weak in the hilus and moderate in the molecular layer (Figures 2A,D,G). The *stratum pyramidale* of the CA1 and CA3 regions and the granule cell layer of the dentate gyrus showed the weakest GluA1–4 immunolabeling (Figures 2A,D,G). The labeling pattern of wild type mice was similar to that of APP/PS1 mice at all ages (Figures 2B,E,H). Quantitative analyses of immunoreactivities performed at the three ages confirmed that the expression levels of GluA1–4 in all subfields and dendritic layers analyzed was unchanged in APP/PS1 mice compared to age-matched wild type controls (Figures 2C,F,I).

We further evaluated the expression of GluA1–4 in the hippocampus using immunoblots of membrane fractions, which reaffirmed the data obtained using histoblots. The guinea pig anti-GluA1–4 antibody revealed one immunoreactive band with estimated molecular mass of ∼105 kDa (Figure 2J). The levels of AMPAR proteins were unchanged in APP/PS1 compared to age-matched wild type mice (Figures 2J,K). Overall, these results indicate no detectable changes in overall expression of AMPAR subunit proteins in APP/PS1 mice brains in any of the investigated brain regions.

### Synaptic AMPAR Distribution Is Unaltered at 1 and 6 Months of Age in APP/PS1 Mice Compared to Wild Type

Using the SDS-FRL method, we analyzed the distribution and densities of AMPARs at excitatory synapses in the CA1 region of hippocampal sections obtained from 1, 6, and 12-months of age wild type and APP/PS1 mice with an antibody against highly conserved extracellular amino acid residues of GluA1–4 (pan-AMPA). We showed that this antibody reacts selectively with all AMPAR subunits GluA1–4. Using the SDS-FRL technique, clusters of IMPs on the E-face represent the postsynaptic membrane specialization (PSDs) of glutamatergic synapses (Tarusawa et al., 2009). Many IMP clusters were labeled with anti-GluA1–4-linked immunogold particles in the CA1 region of the hippocampus.

**FIGURE 3:**
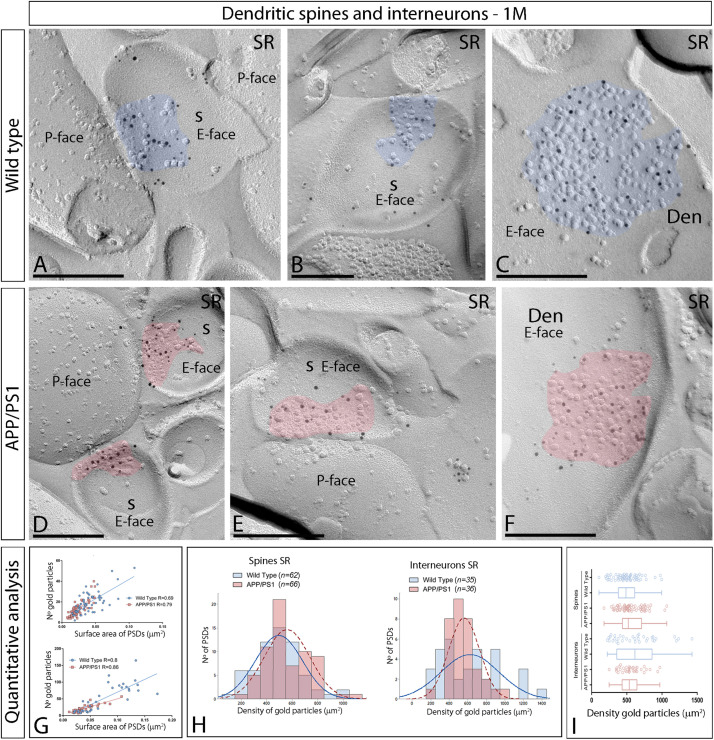
Synaptic AMPARs in dendritic spines and interneurons of APP/PS1 mice at 1 month. **(A–F)** Electron micrographs of the hippocampus showing immunoparticles for AMPARs at excitatory synaptic sites of dendritic spines of pyramidal cells and shafts of interneurons in the *stratum radiatum* of the CA1 region, as detected using the SDS-FRL technique. Postsynaptic membrane specializations (IMP clusters, pseudo colored in blue for wild type and in red for APP/PS1 to aid visualization) show strong immunoreactivity for AMPARs (10 nm gold particles), both in the wild type and in the APP/PS1 mice. Scale bars: **(A–F)**, 200 nm. **(G)** Scatter plots showing the correlation between surface areas of postsynaptic membrane specializations and numbers of gold particles labeling AMPARs in wild type and APP/PS1 mice. **(H)** Histograms showing the distribution of densities of gold particles that label AMPARs of individual postsynaptic membrane specializations in the *stratum radiatum* of the hippocampal CA1 region in wild type and APP/PS1 mice. **(I)** Quantitative analysis showing mean densities of AMPARs in excitatory synapses in spines and interneurons in the *stratum radiatum* in wild type and APP/PS1 mice. No differences were detected in densities of AMPAR immunoparticles in the spines (WT = 496 ± 23 immunoparticles/μm^2^; APP = 560 ± 22 immunoparticles/μm^2^) or interneurons (WT = 626 ± 53 immunoparticles/μm^2^; APP = 562 ± 29 immunoparticles/μm^2^) (Two-way ANOVA test and Bonferroni *post-hoc* test, *p* > 0.05).

**FIGURE 4:**
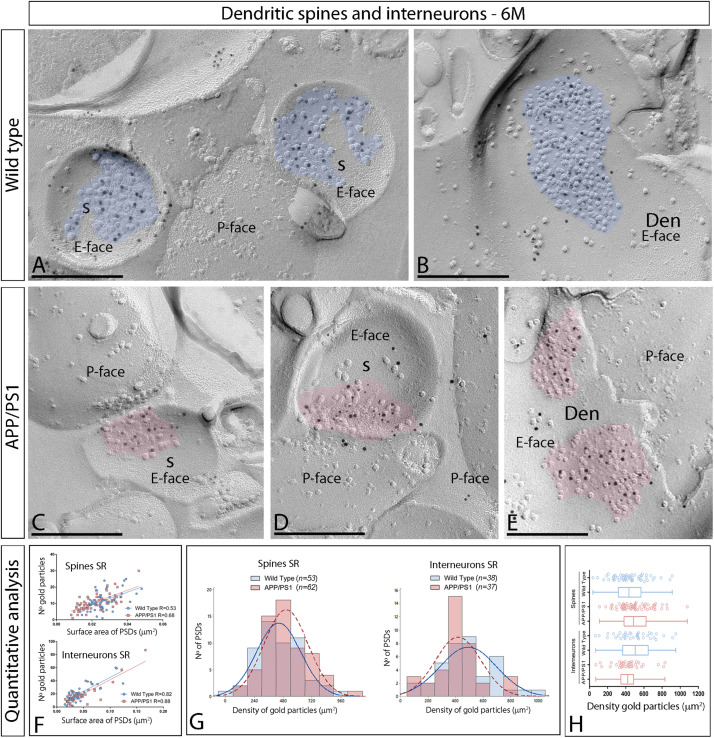
Synaptic AMPARs in dendritic spines and interneurons of APP/PS1 mice at 6 months of age. **(A–F)** Electron micrographs of the hippocampus showing immunoparticles for AMPARs at excitatory synaptic sites of dendritic spines of pyramidal cells and shafts of interneurons in the CA1 *stratum radiatum*, as detected using the SDS-FRL technique at 6 months of age. Postsynaptic membrane specializations (IMP clusters, pseudo colored in blue for wild type and in red for APP/PS1 to aid visualization) show strong immunoreactivity for GluA1–4 (10 nm gold particles) both in the wild type and in the APP/PS1 mice. Scale bars: **(A–E)**, 200 nm. **(F)** Scatter plots showing the correlation between surface areas of postsynaptic membrane specializations and numbers of gold particles labeling AMPARs in wild type and APP/PS1 mice. **(G)** Histograms showing the distribution of densities of gold particles that label AMPARs of individual postsynaptic membrane specializations in the hippocampal CA1 region in wild type and APP/PS1 mice. **(H)** Quantitative analysis showing mean densities of AMPARs in excitatory synapses in spines and interneurons in the *stratum radiatum* in wild type and APP/PS1 mice. No differences were detected in densities of AMPAR immunoparticles in the spines (WT = 443 ± 25 immunoparticles/μm^2^; APP = 505 ± 23 immunoparticles/μm^2^) or interneurons (WT = 494 ± 33 immunoparticles/μm^2^; APP = 421 ± 27 immunoparticles/μm^2^) (Two-way ANOVA test and Bonferroni *post-hoc* test, *p* > 0.05).

We initially performed the ultrastructural analysis at 1 month (Figure 3) and 6 months of age (Figure 4), both in wild type and APP/PS1 mice. In the four experimental groups, immunoparticles for AMPARs were distributed over the entire postsynaptic membrane specializations of spines and interneurons with no apparent clustering (Figures 3A–F, 4A–E). The number of functional AMPARs in individual IMP clusters was plotted against the synaptic area and found to be proportional (Figures 3G, 4F). The density of labeling varied between synapses both in dendritic spines and interneurons (Table 1), but we found no differences between APP/PS1 and WT mice (Figures 3H,I, 4G,H). Overall, these results indicate no changes in the synaptic localization of AMPAR subunit proteins in APP/PS1 mice brains up until 6 months of age.

**TABLE 1:** Number and density of gold particles for pan-AMPAR at different excitatory synapses in the CA1 *stratum radiatum*.

	**1 month**		**6 months**		**12 months**	
	**Spines**	**Interneurons**	**Spines**	**Interneurons**	**Spines**	**Interneurons**
**WT**						
Excitatory synapses (*n*)	62	46	53	38	111	135
Median gold particles	18	53.5	10	18.5	19	22
Range	53–2	165–8	25–1	60–2	81–2	95–3
Density gold particles (μm^2^)						
Mean (±SEM)	496.01 (±23.38)	637.84 (±40.58)	442.89 (±53.27)	494.09 (±33.23)	459.51 (±16.01)	395.70 (±13.70)
Median	487.62	658.88	434.46	504.09	458.96	385.40
Range	995.33–104.97	1235.35–189.89	912.28–38.58	951.47–67.80	883.93–62.44	748.40–98.40
**APP/PS1**						
Excitatory Synapses (*n*)	66	34	62	37	109	173
Median gold particles	13.5	20	12	16	6	10
Range	40–4	57–7	30–1	87–3	39–1	55–1
Density gold particles (μm^2^)						
Mean (±SEM)	560.19 (±22.13)	560.10 (±28.75)	504.60 (±53.27)	420.90 (±27.05)	261.13 (±13.01)	165.78 (±6.94)
Median	520.64	534.57	485.22	421.83	245.31	155.83
Range	1067.81–175.99	969.59–248.82	1077.67–109.78	831.80–69.81	826.88–28.45	462.54–16.57

*Density values are provided in immunogold/μm^2^.*

### AMPAR Content of Excitatory Synapses Is Reduced at 12 Months in APP/PS1 Mice

**FIGURE 5:**
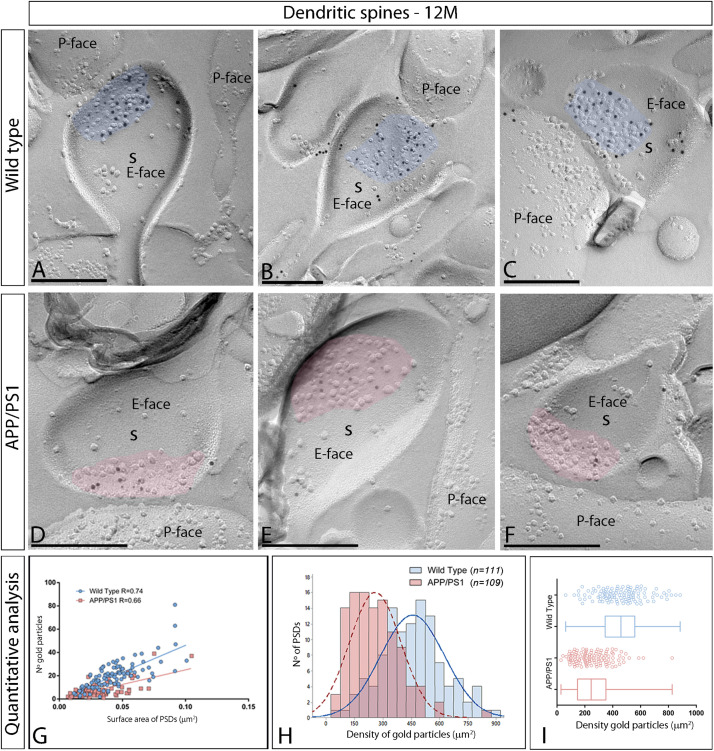
Reduced density of synaptic AMPARs in dendritic spines of APP/PS1 mice at 12 months. **(A–F)** Electron micrographs of the hippocampus showing immunoparticles for AMPARs at excitatory synaptic sites of dendritic spines of pyramidal cells in the CA1 *stratum radiatum*, as detected using the SDS-FRL technique. Postsynaptic membrane specializations (IMP clusters, pseudo colored in blue for wild type and in red for APP/PS1 to aid visualization) show strong immunoreactivity for AMPARs (10 nm gold particles) in the wild type, while they show weaker immunoreactivity in the APP/PS1. Scale bars: **(A–F)**, 200 nm. **(G)** Scatter plots showing the correlation between surface areas of postsynaptic membrane specializations and numbers of gold particles labeling AMPARs in wild type and APP/PS1 mice. **(H)** Histograms showing the distribution of densities of gold particles that label AMPARs of individual postsynaptic membrane specializations in the hippocampal CA1 region in wild type and APP/PS1 mice. **(I)** Quantitative analysis showing mean densities of AMPARs in excitatory synapses in spines in wild type and APP/PS1 mice. A significant reduction in the densities of AMPAR immunoparticles were detected in dendritic spines of APP/PS1 mice (261 ± 13 immunoparticles/μm^2^) compared to age matched wild type (459 ± 16 immunoparticles/μm^2^) (Two-way ANOVA test and Bonferroni *post-hoc* test, *p* < 0.0001).

In the APP/PS1 mouse model of AD severe pathological damage appears at 12 months of age (Garcia-Alloza et al., 2006). In wild type mice, immunoparticles for AMPARs in dendritic spines followed a similar synaptic pattern as at earlier ages, observed over the postsynaptic membrane specializations (Figures 5A–C). In contrast, fewer AMPAR immunoparticles were detected in excitatory synapses of dendritic spines in APP/PS1 mice (Figures 5D–F). The number of gold particles labeling AMPARs was variable (Table 1), but proportional to the synaptic area both in wild type (*R* = 0.74) and APP/PS1 (*R* = 0.66) mice (*p* < 0.0001, Pearson’s correlation test) (Figure 5G). Quantitative analyses revealed a reduction in the number and density of AMPARs in excitatory synapses of dendritic spines. The number of AMPAR immunoparticles distributed in excitatory synapses (*n* = 111) varied in the range of 2–81 with a median value of 19, but in APP/PS1 dendritic spines (*n* = 109) in the range of 1–39 with a median value of 6 (Figure 5H and Table 1). Although the density of labeling varied between synapses (Figure 5I), we found a significant reduction in AMPAR levels in APP/PS1 synapses (median = 245 immunoparticles/μm^2^) compared to age-matched wild type controls (median = 459 immunoparticles/μm^2^) (Two-way ANOVA test and Bonferroni *post-hoc* test, ^****^p < 0.0001) (Figure 5I and Table 1).

**FIGURE 6:**
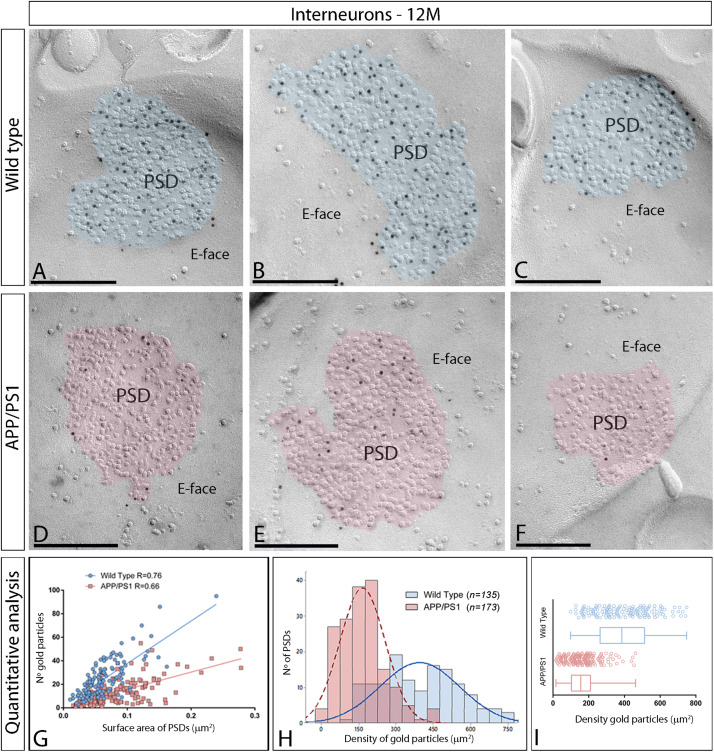
Density of synaptic AMPARs in interneurons of APP/PS1 mice at 12 months. **(A–F)** Electron micrographs of the hippocampus showing immunoparticles for AMPARs at synaptic sites of interneurons in the *stratum radiatum* of the CA1 region, as detected using the SDS-FRL technique. Postsynaptic membrane specializations (IMP clusters, pseudo colored in blue for wild type and in red for APP/PS1 to aid visualization) show strong immunoreactivity for AMPARs (10 nm gold particles) in the wild type, while they show weaker immunoreactivity in the APP/PS1. Scale bars: **(A–F)**, 200 nm. **(I)** Quantitative analysis showing mean densities of AMPARs in excitatory synapses in spines in wild type and APP/PS1 mice. A significant reduction in the densities of AMPAR immunoparticles were detected in synaptic specialization of interneurons in APP/PS1 mice (166 ± 7 immunoparticles/μm^2^) compared to age matched wild type (396 ± 14 immunoparticles/μm^2^) (Two-way ANOVA test and Bonferroni *post-hoc* test, *p* < 0.0001). **(H)** Histograms showing the distribution of densities of gold particles that label AMPARs in SDS-FRL replicas of individual postsynaptic membrane specializations in the hippocampal CA1 region in wild type and APP/PS1 mice. **(G)** Scatter plots showing the correlation between surface areas of postsynaptic membrane specializations and numbers of gold particles labeling AMPARs in wild type and APP/PS1 mice.

Next, we performed ultrastructural analyses to determine whether synaptic AMPARs are also altered in excitatory synapses on interneurons (Figure 6). Similarly to 1 and 6 month old mice, immunoparticles for AMPARs in interneurons at 12 months of age were observed over the postsynaptic membrane specializations in wild type mice (Figures 6A–C), but were consistently fewer in APP/PS1 mice (Figures 6D–F). Although the number of gold particles labeling AMPARs was variable (Table 1), it was proportional to the synaptic area (Figure 6G). In wild type mice, immunoparticles distributed in excitatory synapses (*n* = 135) varied in the range of 3–95 with a median value of 22, but in APP/PS1 dendritic spines (*n* = 173) in the range of 1–55 with a median value of 10 (Figure 6H and Table 1). When density of AMPAR labeling was analyzed, there was a significant reduction of AMPARs in APP/PS1 synapses (median = 156 immunoparticles/μm^2^) compared to age-matched wild type (median = 385 immunoparticles/μm^2^) (Two-way ANOVA test and Bonferroni *post-hoc* test, ^****^p < 0.0001) (Figure 6I and Table 1). In summary, these results indicate a significant decrease in the synaptic localization of AMPAR subunit proteins in APP/PS1 mouse brains at 12 months of age.

### Increase of AMPARs in Intracellular Compartments of Neurons in APP/PS1 Mice

**FIGURE 7:**
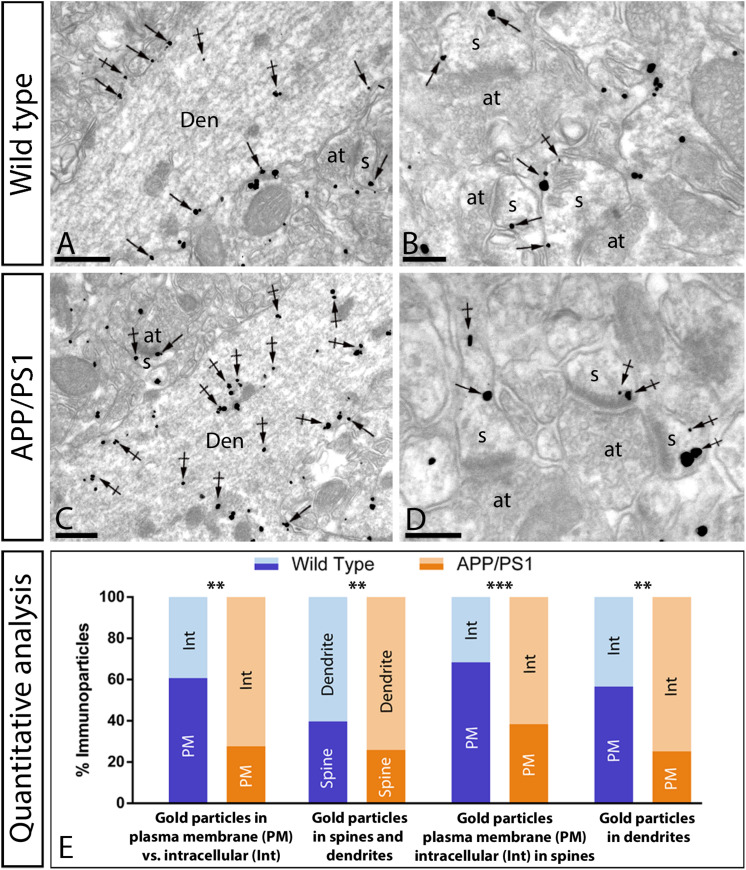
Increased intracellular distribution of GluA2/3 receptors in APP/PS1 mice at 12 months. Electron micrographs showing immunoparticles for GluA2/3 receptors in pyramidal cells of the CA1 region in wild type and APP/PS1 mice, as detected using a pre-embedding immunogold technique. **(A,B)** In wild type mice, immunoparticles for GluA2/3 were found along the extrasynaptic plasma membrane (arrows) and intracellular sites (crossed arrows) of dendritic shafts (Den) and spines (s) contacted by axon terminals (at). **(C,D)** In APP/PS1 mice, immunoparticles for GluA2/3 were more frequently detected at intracellular sites (crossed arrows) and few immunoparticles along the plasma membrane (arrows). **(E)** Quantitative analysis showing that immunoparticles for GluA2/3 receptors were less frequently observed along the extrasynaptic plasma membrane of dendrites and spines of CA1 pyramidal cells in APP/PS1 mice, but instead they more frequently detected at intracellular sites in pyramidal cells. (Student-T test and Holm-Sidak correction, **p < 0.01; ***p < 0.001) Scale bars: **(A,B,D)**, 1 μm; **(C)**, 0.5 μm.

We recently described using the same mouse model that whilst the GABA_B1_ receptor protein expression is not altered, its density is reduced in the plasma membrane and increased at cytoplasmic sites in CA1 pyramidal cells (Martín-Belmonte et al., 2019). To explore whether AMPARs undergo a similar redistribution, their subcellular localization was investigated at 12 months using the quantitative pre-embedding immunogold techniques on tissue blocks taken from the *stratum radiatum* of the CA1 area (Figure 7). The anti-GluA1–4 antibody recognizes extracellular domains of AMPAR subunits, and this prevents the determination of their precise localization, because when two neuronal compartments are close to each other in the neuropil, the origin of immunoparticles cannot always be established unambiguously. Therefore, we used an anti-GluA2/3 antibody raised against the intracellular C-terminal domain of AMPAR subunits, which are exposed on the surface of intracellular compartments and identifiable using immunolabeling (Bernard et al., 1997).

In both wild type and APP/PS1 mice, immunoreactivity for GluA2/3 was detected in dendritic spines and shafts of CA1 pyramidal cells (Figures 7A–D), along their extrasynaptic plasma membrane and also associated at intracellular sites. In wild type mice, most immunoparticles for GluA2/3 were detected along the extrasynaptic plasma membranes of pyramidal cells (Figures 7A,B). In the APP/PS1 mice, however, immunoparticles for AMPARs were more frequently detected intracellularly (Figures 7C,D). This change in localization was demonstrated using quantitative analyses (Figure 7E), which showed significant differences in the total amount of plasma membrane-associated vs. intracellular AMPAR labeling in CA1 pyramidal cells (Plasma membrane: 61 ± 1.9% in wild type and 28 ± 3.6% in APP/PS1; Intracellular: 39 ± 1.9% in wild type and 72 ± 3.6% in APP/PS1; ^∗∗^*p* < 0.01) (Student-T test and Holm-Sidak correction). This change took place both in dendritic spines (Plasma membrane: 68 ± 0.4% in wild type and 38 ± 2.4% in APP/PS1; Intracellular: 32 ± 0.4% in wild type and 62 ± 2.4% in APP/PS1; ^∗∗∗^*p* < 0.001) and dendritic shafts (Plasma membrane: 57 ± 2.6% in wild type and 25 ± 3.5% in APP/PS1; Intracellular: 43 ± 2.6% in wild type and 75 ± 3.5% in APP/PS1; ^∗∗^*p* < 0.01) (Student’s *T*-test and Holm-Sidak correction) (Figure 7E). Given the low expression of GluA2/3 in interneurons compared to pyramidal cells, quantitative analysis was not feasible. To establish the types of intracellular compartments in which AMPARs are present, we performed double labeling immunofluorescence with different marker proteins (Rab4, Rab5 and EEA1), and found no differences in the co-localization pattern in the APP/PS1 mice compared to age-matched wild type controls (Supplementary Figure 3).

## Discussion

In AD, hippocampal dysfunction is produced by synaptic failure (Li and Selkoe, 2020), which leads to memory deficits. Changes in AMPARs are involved in Aβ-related synaptic dysfunction and pathophysiology of AD (*reviewed by* Keifer and Zheng, 2010; Palop and Mucke, 2010; Whitehead et al., 2017). Here, using biochemical and immunocytochemical techniques we examined the expression of AMPARs, and found no change in GluA1–4 protein expression in the APP/PS1 transgenic mouse model of AD. However, using sensitive immunoelectron microscopic techniques to analyze CA1 excitatory synapses in these mice, we identified a reduction of synaptic AMPARs in two different excitatory synapses and a parallel increase in GluA2/3 subunits at intracellular sites. These differences in the density of AMPARs were prominent at 12 months, but not detectable at earlier ages in the APP/PS1 mouse model. These changes likely to be responsible for pathological events in AD.

### Expression of AMPARs in the Hippocampus of APP/PS1 Mice

The expression of AMPARs has been extensively studied in the brain of rodents (Boulter et al., 1990; Keinänen et al., 1990; Petralia and Wenthold, 1992; Sato et al., 1993), and there is substantial knowledge relating to their distribution and regulation during development and aging (Jurado, 2018). In the present study, we have shown by two different techniques that expression levels of AMPARs proteins are not significantly different in APP/PS1 mice with the progressive increase of Aβ levels (Alonso-Nanclares et al., 2013) at different ages in the hippocampus. Our data differs from other studies using different mouse models of AD reporting down-regulation of the protein for AMPAR subunits (Snyder et al., 2005; Baglietto-Vargas et al., 2018; Li et al., 2019).

Previous studies using different techniques in human tissue yielded inconsistent results (Harrison et al., 1990; Dewar et al., 1991; Hyman et al., 1994; Pellegrini-Giampietro et al., 1994; Ikonomovic et al., 1995, 1997; Aronica et al., 1997; Thorns et al., 1997; Wakabayashi et al., 1999; Carter et al., 2004; Ginsberg et al., 2012). Our study identified a clear reduction in AMPAR protein levels in the hippocampus of AD patients (see Supplementary Figure 2), consistent with the reported down-regulation in the expression of GluA1 and GluA2 in AD (Martín-Belmonte et al., 2019). This may be due to the use of different techniques, *post-mortem* delays, brain weight and the age of the subjects, duration of tissue storage, stage of AD or the variability in tissue sampling.

The discrepancy observed between protein expression levels in the mouse model and in human tissue could be the result of differences in brain organization or development of the disease between humans and mice. It is also possible that such differences are due to the fact that the APP/PS1 is an incomplete model of AD. For instance, this model does not develop neurofibrillary tangles, a typical pathologic alteration observed in AD human patients (Irizarry et al., 1997). Alternatively, 12 months old in the APP/PS1 model could be a relatively early stage of pathological evolution to be compared with the stages of AD in human patients with severe AD neuropathology. Despite the unaltered AMPAR protein expression levels in APP/PS1 mice, we explored the possibility that they may still undergo changes in the synaptic localization vs. intracellular sites with AD progression, as also recently described for GABA_B_ receptors (Martín-Belmonte et al., 2019).

### Reduction of Synaptic AMPAR Levels in APP/PS1 Mice: CA1 Pyramidal Cells

While previous studies used the post-embedding immunogold technique to investigate numbers and densities of AMPARs in the hippocampus (Nusser et al., 1998; Petralia et al., 1999; Takumi et al., 1999; Racca et al., 2000), we utilized the highly sensitive SDS-FRL method, with nearly one gold particle-one functional channel sensitivity and proving to be an ideal tool to study the high-resolution subcellular localization of surface-localized molecules (Tanaka et al., 2005). Using this technique, we were able to unravel the distribution of AMPARs in specific CA1 excitatory synapses obtaining data about their density in hippocampal samples displaying AD pathology, at a level of detail and sensitivity never previously attained.

The efficacy of fast glutamatergic neurotransmission in hippocampal neurons depends on the type and number of ionotropic receptors in synapses, particularly AMPARs. Our analysis revealed a great variability in the AMPAR content at individual synapses, similarly to previous reports using post-embedding immunogold techniques (Nusser et al., 1998; Petralia et al., 1999; Takumi et al., 1999; Racca et al., 2000). Consistent with these studies, the number of AMPAR immunoparticles correlated with synaptic area in all experimental groups, but we observed an age-dependent reduction in the density AMPARs in Schaffer collateral synapses in APP/PS1 mice. We detected significant decrease in the synaptic localization of AMPARs and subsequent increase at cytoplasmic sites in CA1 pyramidal cell spines and shafts at 12 months of age, but not earlier.

One important aspect to consider is that the size of individual postsynaptic membrane specializations can affect synaptic transmission. Previous studies have shown that CA1 dendritic spines have PSDs with size around 0.04–0.07 μm^2^ (Schikorski and Stevens, 1997; Shepherd and Harris, 1998). In our work, the average size of PSDs that we found in the *stratum radiatum* of CA1 pyramidal cells in wild type mice at the three ages fell in that range (for instance, 0.046 ± 0.018 μm^2^ at 12 months), and was similar to the size observed previously with similar techniques (Antal et al., 2008). The smaller average size PSDs we encountered in APP/PS1 mice at 12 months is in agreement with previous studies in the same animal model and same age showing a higher proportion of spines with a small head volume in the *stratum radiatum* of the CA1 region (Merino-Serrais et al., 2011). The size of dendritic spines and their PSD are key determinants of synaptic efficacy via control of the distribution of AMPARs (Nusser et al., 1998; Takumi et al., 1999; Matsuzaki et al., 2001).

Interestingly, internalization of AMPARs is required for the induction of LTD (Beattie et al., 2000), a form of synaptic plasticity significantly enhanced in animal models of AD (Shankar et al., 2008). In addition, APP and soluble oligomeric Aβ can induce the removal of surface AMPARs at excitatory synapses, leading to synaptic depression and inhibition of LTP (Walsh et al., 2002; Klyubin et al., 2005; Hsieh et al., 2006). In relation to the effect of the observed unbalance between synaptic and intracellular alterations, the reduction of surface AMPARs and its abnormal trafficking may contribute to the memory deficits in the AD mouse model. This may reflect changes in synaptic plasticity, and this contribute to the memory deficits in the AD mouse model used in this study (Trinchese et al., 2004).

### Reduction of Synaptic AMPARs in APP/PS1 Mice: Interneurons

Most studies of AD brains have concentrated on excitatory neuronal functions, but compelling studies also implicate impaired inhibition in the pathogenesis (Palop and Mucke, 2010; Verret et al., 2012; Hazra et al., 2013; Martinez-Losa et al., 2018). Our results demonstrate a significant reduction in the synaptic localization of AMPARs in excitatory synapses onto dendritic shafts of interneurons in the APP/PS1 mouse model, consistent with past evidence that interneurons and the oscillatory network activities they regulate are altered in AD (Palop and Mucke, 2016). Given the random fractures in our replica samples (Masugi-Tokita and Shigemoto, 2007), dendritic shafts of interneurons could not be followed back to the parent cell body. Therefore, we could not exclude the possibility that dendritic shafts establishing excitatory synapses with different AMPAR contents represent dendrites originating from distinct interneurons or just the same interneuron receiving distinct inputs. Technical limitations prevented unequivocal identification of the source of dendritic shafts, but since the region of *stratum radiatum* we analyzed was close to pyramidal cell bodies, these dendrites are likely to arise from PV-positive cells (Nyíri et al., 2003), which represent basket and axo-axonic cells whose axon arborizations target the soma and the axon initial segment of principal cells, respectively (Freund and Buzsáki, 1996; Klausberger and Somogyi, 2008).

Upon *post mortem* examination, the hippocampi of AD patients show a reduction in the number of PV-expressing interneurons, as well as other subpopulations, in the hippocampus (Brion and Résibois, 1994; Brady and Mufson, 1997). The significant reduction of synaptic AMPARs described here in interneurons would lead to an aberrant weakening of excitatory synapses, thus contributing to hyperexcitability and deficits in synchrony. Interestingly, weakening of synapses due to the reduction of AMPAR currents has been involved in the impairment of network oscillatory activity (Caputi et al., 2012), which is known to be essential for learning and memory. This too contributes to cognitive abnormalities in AD (Verret et al., 2012; Palop and Mucke, 2016). Therefore, improving the function of interneurons may be of therapeutic benefit in AD.

In summary, this study is the first to report that reduction of AMPARs takes place in excitatory synapses established on both pyramidal cells and interneurons in APP/PS1 mice. Reductions in the receptors at synapses in both pyramidal cells and interneurons are likely to contribute to the memory deficits associated with AD. A deeper understanding of the subcellular localization of AMPARs in the neurobiology of AD may elucidate further mechanisms of the disease.

## Data Availability Statement

The raw data supporting the conclusions of this article will be made available by the authors, without undue reservation, to any qualified researcher.

## Ethics Statement

The studies involving human participants were reviewed and approved by the Brain Bank of the Institute of Neuropathology of the University Hospital Bellvitge IDIBELL (HUB-ICO-IDIBELL-Biobank). The patients/participants provided their written informed consent to participate in this study. The animal study was reviewed and approved by the Comité Ético de Experimentación Animal (CEEA) UCLM.

## Author Contributions

RL and YF designed the project. MI developed the rabbit anti-GluA1–4 (D160, pan-AMPA) receptor polyclonal antibody. AM-B, RL, and YF performed SDS-FRL immunoelectron microscopy. AM-B, CA, and AM-M performed histoblot analysis. AM-B performed western blots. AM-B, RA-R, and AM-M performed pre-embedding immunoelectron microscopy and its quantitative analysis. LO developed in-house software and performed computational analysis. EM provided reagents, training in the histoblot method, and feedback on the analysis. AM-B, RA-R, CA, and RL analyzed the data. RL wrote the manuscript. All authors read and approved the final manuscript, had full access to all data in the study and take responsibility for the integrity of the data and the accuracy of the data analysis.

## Conflict of Interest

EM is Scientific Advisory Board member of Hello Bio (http://www.hellobio.com).

The remaining authors declare that the research was conducted in the absence of any commercial or financial relationships that could be construed as a potential conflict of interest.

## Footnotes

1 https://www.jax.org/strain/005864

2 https://www.alzforum.org/research-models/
