# Supplementary material for: Age-Dependent Shift of AMPA Receptors From Synapses to Intracellular Compartments in Alzheimer’s Disease: Immunocytochemical Analysis of the CA1 Hippocampal Region in APP/PS1 Transgenic Mouse Model
Source: Front Aging Neurosci. 2020 Oct 6;12:577996. doi: 10.3389/fnagi.2020.577996 (PMC7572859; doi:10.3389/fnagi.2020.577996)
Supplement: Supplementary file 1 [file Data_Sheet_1.docx]

**Supplementary material**

**Age-dependent shift of AMPA receptors from synapses to intracellular compartments in Alzheimer´s disease: Immunocytochemical analysis of the CA1 hippocampal region in APP/PS1 transgenic mouse model**

Alejandro Martín-Belmonte, Carolina Aguado, Rocío Alfaro-Ruíz, Makoto Itakura, Ana Esther Moreno-Martínez, Luis de la Ossa, Elek Molnár, Yugo Fukazawa and Rafael Luján

**Supplementary Material & Methods**

***Production and characterisation of the pan-AMPAR antibody***

**Plasmids.** Full-length rat GluA1, GluA2 and GluA4 cDNAs were subcloned into pcDNA3 (plasmid vector, Invitrogen) to generate pc3-GluA1, pc3-GluA2 and pc3-GluA4, respectively. In addition, full-length murine GluA3 cDNAs was subcloned into pRK5 (plasmid vector) to generate pRK5-GluA3.

**Transfection, immunoprecipitation and immunoblot analysis.** HeLa cells were maintained in Dulbecco's modified Eagle's medium (DMEM) with 10% fetal bovine serum (FBS) and were transiently transfected with GluA1-4 expression plasmids using Lipofectoamine 2000 (Invitrogen). After 48 h of transfection, the cultured cells were harvested and lysed in 0.5% Triton X‐100 in 20 mM Tris/HCl and 50 mM NaCl (pH 7.5). For immunoprecipitation experiments, anti‐AMPAR antibodies (0.75 μg protein) were bound to 5 μL of Protein G Dynabeads. The transfected HeLa cell lysates were incubated with the antibody‐conjugated Protein G Dynabeads. Beads were washed three times with 0.5% Triton X‐100 in 20 mm Tris/HCl and 50 mm NaCl (pH 7.5), and the immunoprecipitated materials were recovered in SDS sample buffer (2% SDS, 3.3% glycerol, 125 mm Tris/HCl, pH 7.4) heated at 90 °C for 5 min. The samples were separated by SDS-PAGE, followed by electrotransfer into PDVF membrane and incubated with pan-GluA1-4 AMPA receptor antibody (D160) and (C095). Following labelling with HRP-conjugated secondary antibody, immunopositive bands were detected by the enhanced chemiluminiscence method using a commercially available kit (Nakarai Chemicals).

***Human samples***

Human brain tissue was obtained at autopsy from the Pathologic Anatomy Service of Bellvitge University Hospital (Barcelona, Spain), following national laws and international ethical and technical guidelines on the use of human samples for biomedical research purposes. Tissue blocks obtained from the hippocampal formation of eight individuals were studied (**Table 1**). Four had clinical and *post mortem* neuropathological diagnoses of AD defined according to (Braak and Braak, 1991) (**Table 1**). Four were non-demented subjects who did not meet the neuropathological criteria of AD and were therefore classified as control cases (**Table 1**). All hippocampal samples were obtained from 3 to 8.5 h after death and were immediately prepared for biochemical studies using immunoblots, and coded for blinded experiments. All tissue samples were frozen on dry ice or in liquid nitrogen at time of autopsy and stored at −80 ºC until used for analysis. Samples were decoded following the completion of experiments and prior to data analysis. Additional clinical data for the *post-mortem* brains used were not available from the brain bank.

**Supplementary Results**

***Production and specificity of a new rabbit anti-GluA1-4 (pan-AMPAR) antibody***

The rabbit polyclonal antibody against GluA1-4 (pan-AMPAR, D160) was newly developed in this study by following previously established protocol (Nusser et al., 1998; Pickard et al., 2000) and this antibody was used for SDS-FRL experiments. The reactivity of this antibody for AMPAR subunits was examined by immunoblot analysis with heterologously expressed GluA1-4 subunit proteins. This new pan-AMPAR antibody recognized all four AMPA receptor subunits (**Supplementary Fig. 1**). In this experiment, we also confirmed the reactivity of guinea pig anti-AMPAR antibody (C095) against GluA1-4 subunit proteins.

***There is a reduction in AMPARs expression in patients with Alzheimer´s disease***

Because previous studies (Ikonomovic et al., 1995, 1997; Aronica et al., 1997; Thorns et al., 1997; Wakabayashi et al., 1999) reported changes in individual AMPAR subunits in AD, we assessed the total expression levels of all AMPAR proteins (GluA1-4) in whole tissue lysates of the hippocampus in control and AD cases using immunoblot (**Supplementary Fig. 2**). In total hippocampal homogenates of all cases, we detected GluA1-4 as a single predominant band at ~105 kDa (**Supplementary Fig. 2A**). Quantification of this immunoreactive band revealed that GluA1-4 proteins were significantly reduced in the hippocampus of AD subjects compared to controls (**Supplementary Fig. 2B**).

***Co-localization between GluA1-4 and protein makers***

To establish the types of intracellular compartments in which AMPARs are present, we performed double labelling immunofluorescence with different marker proteins: Rab4 (small G protein), a marker of early endosomes; Rab5 (small GTPase), a marker of early endocytic pathway; and EEA1 (early endosomal antigen 1), a marker or early endosomes and amphisomes (**Supplementary Fig. 3**). Our analysis focused in the somata and proximal dendrites of CA1 pyramidal cells. Immunoreactivity for GluA2/3 was detected as punctate through the cytoplasm surrounding the nuclei of pyramidal cells (**Supplementary Fig. 3A1-F1**). Immunoreactivity for Rab4 (**Supplementary Fig. 3A2, B2**), Rab5 (**Supplementary Fig. 3C2, D2**) and EEA1 (**Supplementary Fig. 3E2, F2**) were also detected as punctate labelling through the cytoplasm. We observed co-localisation between GluA2/3 and the different marker proteins (**Supplementary Fig. 3A3-F3**). We then measured the total area of labelling and area of co-localisation to obtain the percentage of co-localisation for each maker. This quantitative analysis showed no differences in the percentage of co-localisation in APP/PS1 mice compared to age-matched wild type controls with the makers employed (Rab4: 0.0009 ± 0.00009% in WT and 0.0009 ± 0.00021% in APP/PS1; Rab5: 0.0012 ± 0.0001% in WT and 0.0012 ± 0.0002% in APP/PS1; EEA1: 0.0011 ± 0.0002% in WT and 0.0011 ± 0.0001% in APP/PS1). No statistical significance was found using the student-t test with the Holm-Sidak correction (**Supplementary Fig. 3A3-F3**).

**References**

Aronica, E., Dickson, D. ., Kress, Y., Morrison, J. ., and Zukin, R. . (1997). Non-plaque dystrophic dendrites in Alzheimer hippocampus: a new pathological structure revealed by glutamate receptor immunocytochemistry. *Neuroscience* 82, 979–991.

Braak, H., Braak, E., 1991. Neuropathological stageing of Alzheimer-related changes. Acta Neuropathol. https://doi.org/10.1007/BF00308809

Ikonomovic, M. d., Mizukami, K., Davies, P., Hamilton, R., Sheffield, R., and Armstrong, D. M. (1997). The Loss of GluR2(3) Immunoreactivity Precedes Neurofibrillary Tangle Formation in the Entorhinal Cortex and Hippocampus of Alzheimer Brains. *J. Neuropathol. Exp. Neurol.* 56, 1018–1027. doi:10.1097/00005072-199709000-00007.

Ikonomovic, M. D., Sheffield, R., and Armstrong, D. M. (1995). AMPA-Selective glutamate receptor subtype immunoreactivity in the hippocampal dentate gyrus of patients with Alzheimer disease. *Mol. Chem. Neuropathol.* 28, 59–64. doi:10.1007/BF02815205.

Nusser, Z., Lujan, R., Laube, G., Roberts, J. D. B., Molnar, E., and Somogyi, P. (1998). Cell type and pathway dependence of synaptic AMPA receptor number and variability in the hippocampus. *Neuron* 21, 545–559. doi:10.1016/S0896-6273(00)80565-6.

Thorns, V., Mallory, M., Hansen, L., and Masliah, E. (1997). Alterations in glutamate receptor 2/3 subunits and amyloid precursor protein expression during the course of Alzheimer’s disease and Lewy body variant. *Acta Neuropathol.* 94, 539–548. doi:10.1007/s004010050748.

Wakabayashi, K., Narisawa-Saito, M., Iwakura, Y., Arai, T., Ikeda, K., Takahashi, H., et al. (1999). Phenotypic down-regulation of glutamate receptor subunit GluR1 in Alzheimer’s disease. *Neurobiol. Aging* 20, 287–295. doi:10.1016/S0197-4580(99)00035-4.

**TABLE 1**. Summary of the main clinical and neuropathological data in the present series.

| *Case Nº* | Gender | Age | PMD (h) | Braak stage | CERAD |
| --- | --- | --- | --- | --- | --- |
| *1* | Male | 53 | 6.15 | I | 0 |
| *2* | Male | 74 | 4.0 | I | 0 |
| *3* | Male | 72 | 8.45 | II | A |
| *4* | Female | 66 | 3.15 | II | A |
| *5* | Male | 74 | 6.0 | V | C |
| *6* | Female | 89 | 5.0 | VI | C |
| *7* | Female | 80 | 4.15 | VI | C |
| *8* | Male | 78 | 5.45 | V-VI | C |

Braak Stages (Braak and Braak, 1991): I–II (NFTs in entorhinal cortex and closely related areas); III–IV (NFTs abundant in amygdala and hippocampus and extending slightly into the association cortex); V–VI (NFTs widely distributed throughout the neocortex and ultimately involving primary motor and sensory areas). CERAD Stages (Fillenbaum et al., 2008): A, low density of neuritic plaques; B, intermediate density of neuritic plaques; C, high density of neuritic plaques. PMD, post-mortem delay; CERAD, Consortium to Establish a Registry for Alzheimer's Disease.

**SUPPLEMENTARY FIGURES**

**SUPPLEMENTARY FIGURE 1**


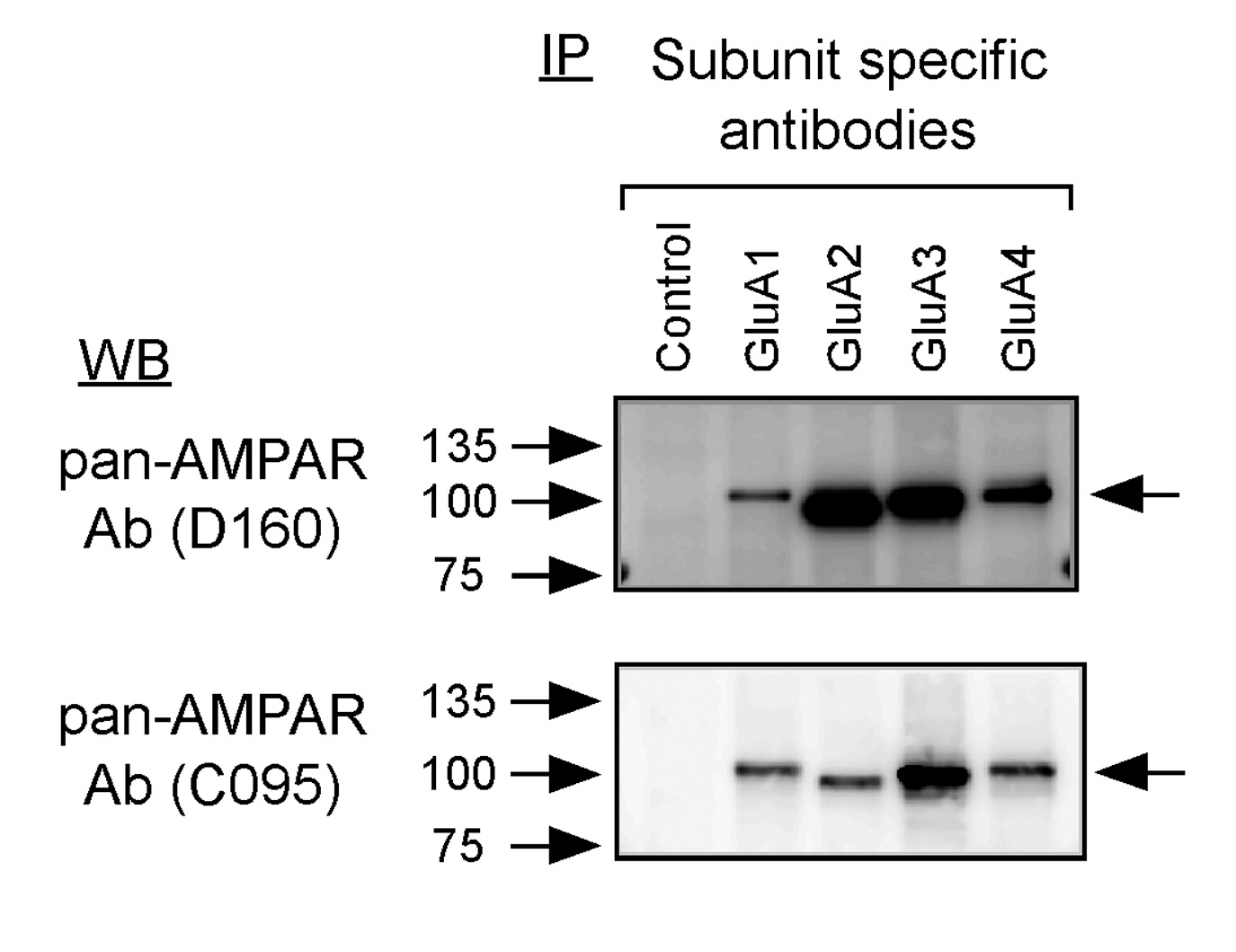


**Supplementary Figure 1**. ***Characterisation and reactivity of anti-GluA1-4 (pan-AMPAR) antibody (D160) using immunoblots***. HeLa cells were transfected with expression vectors for AMPAR subunits (GluA1-4). Transfected cell lysates were immunoprecipitated with subunit-specific antibodies and then immunoblotted with anti-GluA1-4 (pan-AMPAR) antibodies (D160 and C095). Both antibodies reacted with all 4 AMPAR subunit proteins (GluA1-4).

**SUPPLEMENTARY FIGURE 2**


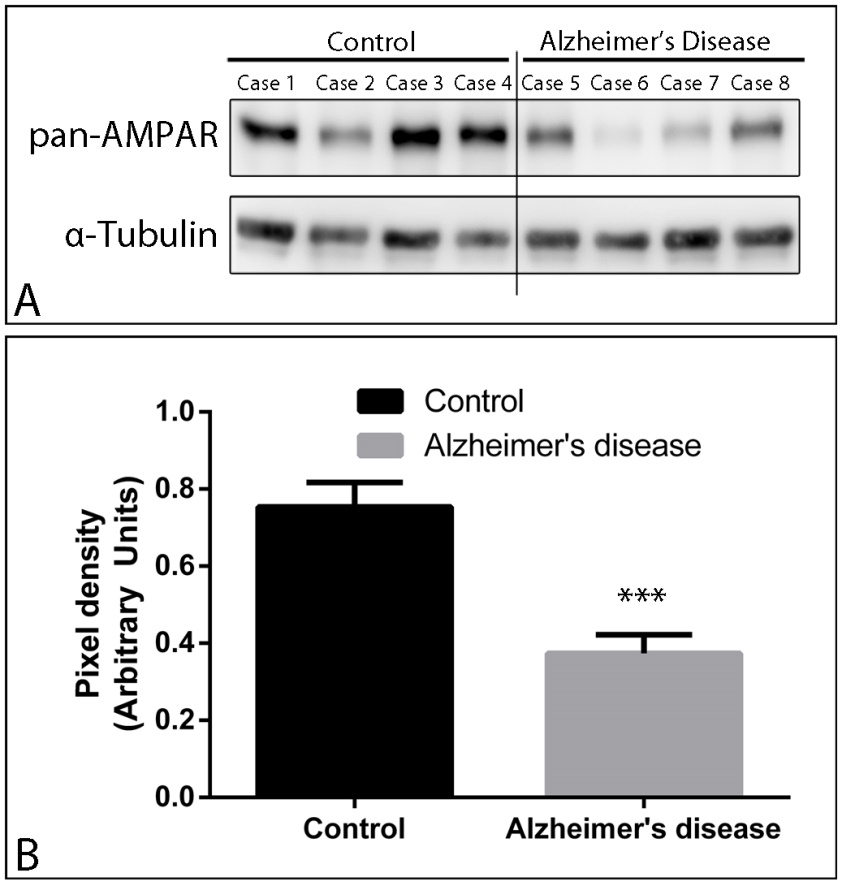


**Supplementary Figure 2. *Representative immunoblots of AMPARs in the hippocampus from control and Alzheimer´s patients***. (A) Crude membrane preparations were subjected to 7.5% SDS-PAGE, transferred onto polyvinylidene difluoride membranes. Samples were obtained from separate individuals without and with AD symptoms (see Table 1). They were reacted with the pan-AMPAR antibody, which identified a single predominant band with estimated molecular mass of 100 kDa. (B) The developed immunoblots were scanned and densitometric measurements were averaged to compare the immunoreactivities in the hippocampal regions of control and AD brains. Quantification of GluA1-4 immunoreactivities were normalised to the α-tubulin content of the relevant sample and expressed as pixel density showed a significant reduction in the amount of protein when compared AD with controls. Data are means ± SEM of represented cases. ***p<0.001; mean difference of 0.38; IC95% (0.22-0.54).

**SUPPLEMENTARY FIGURE 3**


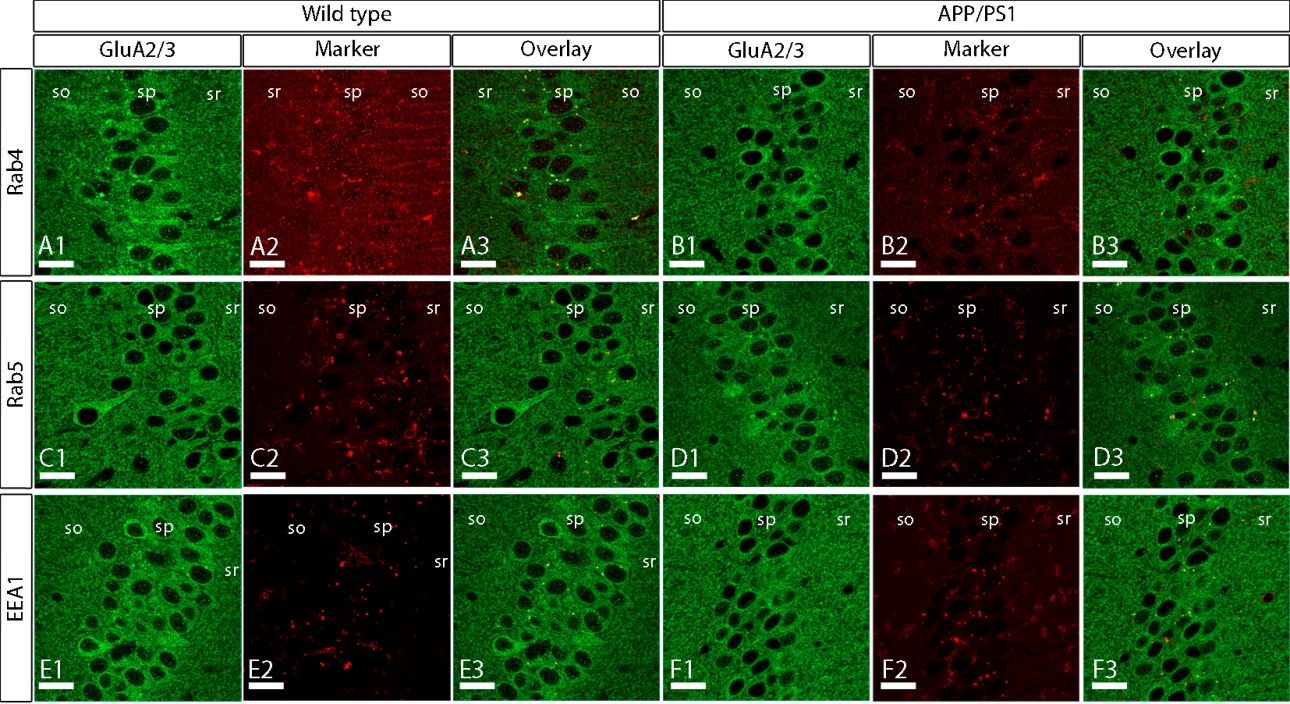


**Supplementary Figure 3**. ***Co-localisation of GluA2/3 receptors with marker proteins*** ***in APP/PS1 mice at 12 months***. (A-F) Immunofluorescence for GluA2/3 (green) and Rab4 (red), Rab5 (red) and EEA1 (red) in wild type and APP/PS1 mice. The overlay between GluA2/3 and each marker protein can be seen in yellow (A3-F3). Co-localisation between GluA2/3 and the different marker proteins was detected mainly in the somata of pyramidal cells in the *stratum pyramidale*, but no differences in the frequency of co-localisation was observed in the APP/PS1 mice compared to age-matched controls (Rab4: 0.0009% ± 0.00009 in WT and 0.0009% ± 0.00021 in APP/PS1; Rab5: 0.0012% ± 0.0001 in WT and 0.0012% ± 0.0002 in APP/PS1; EEA1: 0.0011% ± 0.0002 in WT and 0.0011% ± 0.0001 in APP/PS1). No statistical significance was found using the student-t test with the Holm-Sidak correction. so, *stratum oriens*; sp, *stratum pyramidale*; sr, *stratum radiatum*. Scale bars: A-F, 20 µm.
